# Annotations

**Published:** 1895-07-20

**Authors:** 


### ANNOTATIONS.

### The Scientific Manufacture of Poets.

SCIENCE, in the persons of Lombroso and Nordau, tells us exactly how poets and other outstanding geniuses have been made in the past. Mr. Grant Allen, in a very amusing skit, does his best to anticipate for us the definite application of scientific principles to the manufacture of poets on a large and varied scale, which the coming centuries of our era are likely to witness. By the use of a process which will, no doubt, soon be quite easy to advanced scientists, he finds himself "in the spirit" in the twenty-first century. In the laboratory of a distinguished Germano-Semitic scientist and philosopher he becomes an eye-witness of the actual process of poet-manufacture. It is all a case of heredity. There is no need to go back further than the grandparents. But even a poet must have grandparents, and not fewer than four of them. Accordingly, for one grandparental pair "a very fine inebriate" is united to a "lovely case of advanced hysteria," and for the other pair of grandparents "an opium-eating doctor" is married to a "bishop's daughter who has taken to theosophy." By the use of the occult process which has become a part of science in the twenty-first century we are hurried through the interesting period of fifty or sixty years, and introduced to the "divine poet" who is the ultimate offspring of this double set of grandparents. He is found in a hospital ward, dying of phthisis; and the Germano-Semitic scientist is eagerly awaiting the verses which he is destined by heredity to produce. Somewhat anxiously the scientist asks the nurse whether her patient, who is now twenty-two years of age and should have produced several octavos, has yet written very much verse. "Not a line of verse," as yet, the nurse informs him; but he has invented an admirable machine for neatly and expeditiously "drawing the corks of champagne bottles." The fooling is very clever—quite in Mr. Grant Allen's best style—and we commend it to the consideration of all those would-be men of science who, out of an ounce of fact and many pounds of crazy hypothesis, construct theories which in our forefathers' days no one out of Bedlam would have taken the trouble either to construct, or to read, or to reply to.

### Clinical Research.

THE whole medical profession has had a guiding and a stimulating hand held out to it by the establishment of the Clinical Research Association. In the days of studentship every third and fourth year's man is a pathologist, a diagnostician, and, in intention at any rate, a scientific physician or surgeon. Such he still strives to be when he has entered upon practice for himself. But the practical difficulties of getting secretions and excretions trustworthily examined, and the almost impossibility of obtaining confirmations of diagnosis by post-mortem examinations, gradually bring about a feeling of *non possumus* in the average medical man's mind, and he learns at length to be content with being merely a more or less skilful therapeutician. It is an excellent thing to be a good therapeutician; but it is still better to be a careful and progressive man of science as well. The mere therapeutician may serve himself, and be of great use to his own immediate patients; but the scientific observer and recorder, though but a village practitioner, may at some time or other render services to the whole of medicine and the world. The Clinical Research Association makes it possible for every young practitioner to begin and continue the practice of his profession on scientific lines. He can now diagnose lung diseases, kidney diseases, and manifold other pathological conditions with absolute certainty by the aid of the society's examinations. He can, moreover, make much use of what may be called "limited post-mortems." Many relatives will allow of the removal of a kidney, or a portion of lung, or liver, or brain, who would not think of consenting to a complete post-mortem examination of their dead. In short, if the Clinical Research Association does but show adequate business capacity in recommending itself to the profession, and if the profession on its part will but learn how to utilise the society in its widest and most practical sense, we may almost say that a new scientific era has dawned for the hard-working general practitioner. Every man in medicine who has not already done so should write to 1, Southwark Street, London Bridge, for the Association's prospectus and programme.

### Medical Politicians.

THE General Election now in progress reminds the medical profession once more of its powerlessness as a political force. Whilst lawyers are numbered by scores, almost by hundreds, in the House of Commons, and bishops constitute a most significant element in the House of Lords, medical men, who have never yet got beyond the Commons, might, even in the people's House, almost be counted on the fingers of one hand. This is seriously injurious to a country which is becoming increasingly crowded, increasingly worried by every variety of physiological pressure, and increasingly in need of physiological guidance of the highest and most trusted order. In politics doctors are, and always have been, "a feeble folk." The question for us is this: Can nothing be done to give us our right place of influence and authority in the councils of the nation? Apparently not. To the lawyer a seat in Parliament means increasing work and more profitable wages; to the practising doctor it means the loss of both wages and work. The practising doctor, therefore, of necessity, fights shy of Parliament. There are, however, some medical men who could afford to forego further wealth, and who might, with great advantage to their profession and the public, give themselves up to a Parliamentary career. But it is to be feared that most of such men are well past middle life. Moreover, the ways of the aged doctor are not Parliamentary ways. A man who has been thirty or forty years laying down the law to his patients, and whose word has been accepted as an oracle in hundreds of admiring households, could not, without very great difficulty, bring himself to endure the rough and tumble of Parliamentary life. Still, if and when London is provided with one great medical school positions may then be found for younger men who could with advantage enter Parliament, and so secure a fairer representation of medical opinion.

